# An internal hernia caused by Meckel’s diverticulum: a case report

**DOI:** 10.1186/s12876-020-01211-4

**Published:** 2020-03-12

**Authors:** Yang Zhang, Yuchen Guo, Yuanlin Sun, Yuechao Xu

**Affiliations:** Department of Gastrointestinal Surgery, First Hospital of Jilin University, Changchun, 130021 Jilin China

**Keywords:** Meckel’s diverticulum, Intestinal obstruction, Hernia, Laparotomy, Case report

## Abstract

**Background:**

Meckel’s diverticulum is a remnant of the omphalomesenteric duct. It can lead to intestinal perforation, obstruction and gastrointestinal bleeding. While the internal hernia caused by Meckel’s diverticulum is rarely reported.

**Case presentation:**

We report a case of a 45-year old female patient who presented with intestinal obstruction and on laparotomy was found to have Meckel’s diverticulum with internal hernia causing intestinal gangrene. Segmental bowel resection was performed and the patient had uneventful recovery.

**Conclusions:**

In patients with acute intestinal obstruction without previous abdominal surgery, Meckel’s diverticulum and its complications should be suspected.

## Background

Internal hernia refers to the displacement of the abdominal internal organs from their original position through a normal or abnormal channel or fissure in the abdominal cavity to an abnormal cavity. The internal hernia is one of the uncommon causes of acute abdomen. They are often secondary to abdominal adhesions or iatrogenic mesenteric defects caused by previous abdominal surgery. A small number of patients without previous abdominal surgery can develop internal hernia such as paraduodenal hernia, hernia through Foramen of Winslow. However, it is extremely rare for the Meckel’s diverticulum to cause internal hernia leading to intestinal gangrene. This paper reports a case of internal hernia with intestinal obstruction and gangrene caused by Meckel’s diverticulum.

## Case presentation

**Fig. 1:**
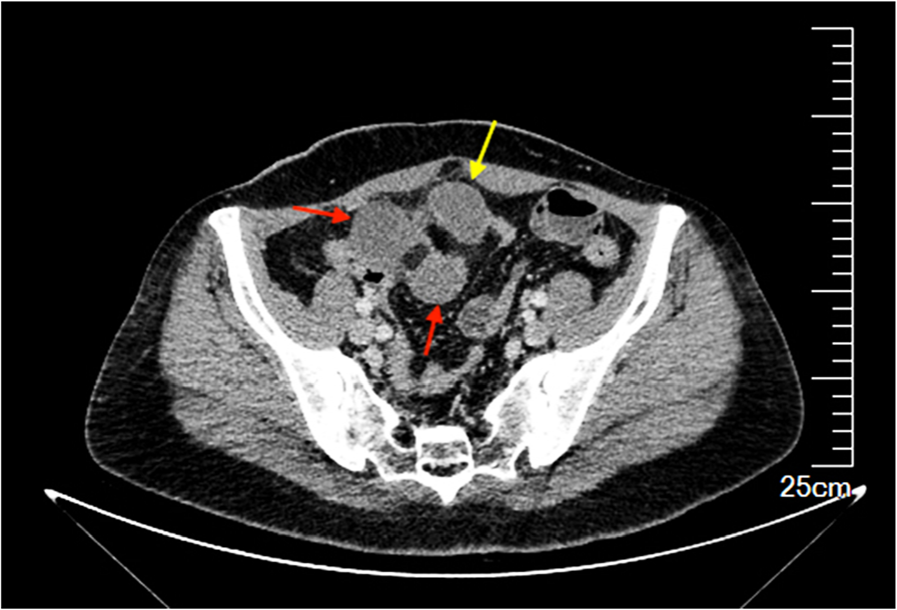
Computed tomography showing the thickened, dilated small bowel loop (red arrow) with surrounding stranding formed by Meckel diverticulum (yellow arrow)

A 45-year old female patient presented with complaints of abdominal pain accompanied by nausea and vomiting for 1 day. The body mass index (BMI) of the patient was 22.86 kg/m^2^. She had no previous medical or surgical illness. On admission, her pulse rate was 101 beats per minute, blood pressure was 95/65 mmHg, and body temperature was 38.4 °C. On physical examination, there was abdominal distension, periumbilical tenderness and rebound tenderness. Preoperative laboratory examinations revealed white blood cell count of 15.54 × 10^9^/L (normal range 3.5 × 10^9^/L -9.5 × 10^9^/L). C-reactive protein was 24.07 mg/L (normal range 0-3 mg/L). Contrast enhanced computed tomography revealed focal dilatation and thickening of the small bowel loop with surrounding fat stranding raising possibility of intestinal obstruction due to internal herniation (Fig. 1). The provisional diagnosis of strangulated intestinal obstruction was made. After initial resuscitation, the patient underwent exploratory laparotomy.

**Fig. 2:**
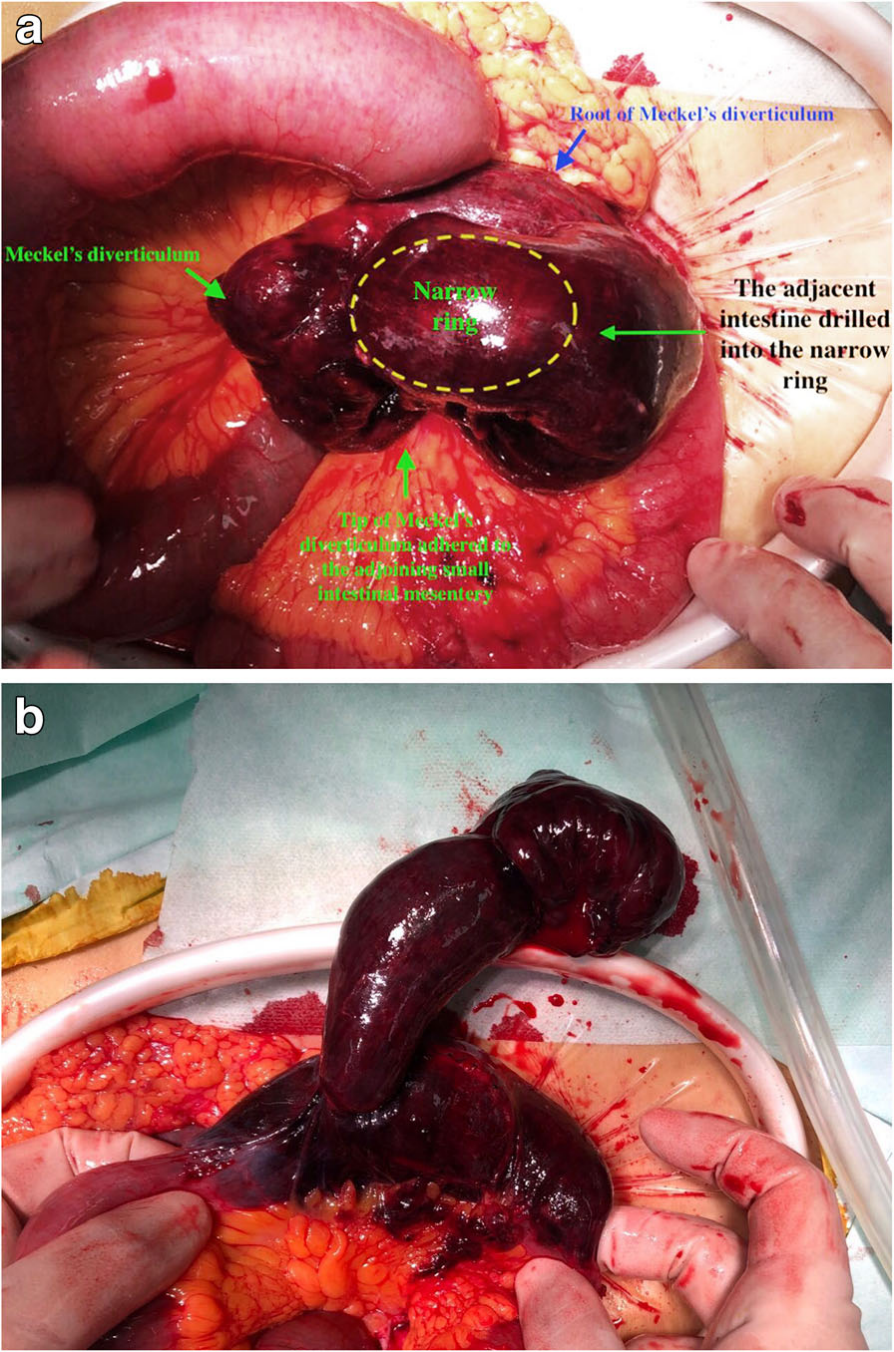
Intraoperative photographs showing the tip of 5 cm long Meckel’s diverticulum adhered to the adjoining small intestinal mesentery (**a**). A part of the adjacent intestine drilled into the narrow ring formed by the diverticulum and mesentery. After the adhesiolysis, the herniated intestine was found to have ischemic necrosis (**b**)

At surgery, a 5-cm-long Meckel’s diverticulum was found one meter away from the ileocecal junction. The tip of the diverticulum was densely adhered to the adjoining mesentery forming a narrow ring. A part of the small intestine was seen drilled into the narrow ring formed by the diverticulum and mesentery causing gangrene of the herniated small intestine (Fig. 2). Adhesiolysis and resection of the Meckel’s diverticulum with the gangrenous bowel with anastomosis was performed. The postoperative course was uneventful. The patient was discharged from the hospital on the fifth postoperative day. At 3-month postoperative follow-up, the patient is symptom-free and has restored normal activity and diet.

## Discussion and conclusions

Internal hernias account for up to 6% of the patients with intestinal obstruction [1, 2]. Complications due to internal hernias often require emergency abdominal surgery which is associated with significant morbidities and mortality. The most frequent manifestations include persistent progressive abdominal crampy pain, nausea, vomiting and abdominal distension. However, in internal hernias, the early abdominal signs tenderness, guarding are minimal in comparison to the severity of the symptoms. By the time patient develops, obvious signs of peritonitis such as rebound tenderness and rigidity, most of them have intestinal gangrene and intestinal perforation. Therefore, early diagnosis is the key in the management of Intra-abdominal internal hernias.

Most of the internal hernias occur secondary to abdominal surgery and primary internal hernia is relatively rare. The main causes of internal primary hernia include paraduodenal hernias, Meckel’s diverticulum, chronic appendicitis, obturator hernia, mesenteric defects, sigmoid crypt sac, omentum and mesenteric adhesions. In the index case, adhesions between Meckel’s diverticulum and the mesentery led to the development of narrow ring precipitating internal hernia.

Meckel’s diverticulum is the most common digestive tract malformation. During the fifth to seventh weeks of embryonic development, the vitelline duct degenerates and becomes atretic. Any abnormality during this process can lead to development of umbilical intestinal fistula, umbilical sinus, vitelline duct cyst or Meckel’s diverticulum. Meckel’s diverticulum occurs in about 2 to 3% of the population, with similar rates in men and women [3, 4]. It is usually found in the small intestine 30 cm to 150 cm away from the ileocecal region. In most cases, Meckel’s diverticulum does not cause significant gastrointestinal symptoms, however, about 4% of patients with this digestive tract malformation develop complications including gastrointestinal bleeding, diverticulitis, perforation and intestinal obstruction [3, 5, 6]. In some rare cases, Meckel’s diverticulum may be incarcerated into the inguinal canal or femoral hernia, or other weak point of the abdominal cavity, which is also referred to as Littre hernia [7, 8].

There are several reasons for development of intestinal obstruction due to Meckel’s diverticulum. First, repeated inflammation can lead to the formation of adhesion bands between diverticulum and the abdominal wall or mesentery which can cause adhesive obstruction. Second, the remnant of omphalomesenteric duct in the form of adhesion band can persist connecting the Meckel’s diverticulum to the umbilicus. This connecting band can precipitate intestinal torsion leading to obstruction. Third, Meckel’s diverticulum can act as a lead point for the development of intussusception and intestinal obstruction. Fourth, the diverticulum can act as a nidus for bezoar formation which can cause intestinal obstruction. Occurrence of internal hernia and intestinal gangrene due to Meckel’s diverticulum is rare [9, 10]. Therefore, in young and middle-aged patients without previous abdominal surgery, if acute intestinal obstruction occurs, internal hernia and Meckel’s diverticulum should be included in the differential diagnosis. Early preoperative diagnosis is of great significance for treatment.

Preoperative diagnosis of Meckel’s diverticulum is usually difficult. In most of cases, it is found during surgery. In some patients with incomplete intestinal obstruction, water soluble oral contrast study can help in making the diagnosis. However, for patients with complete obstruction, abdominal CT examination is of great significance for definitive diagnosis as seen in the current case.

Surgical excision is the treatment of choice for Meckel’s diverticulum. Surgery can be done laparoscopically or by laparotomy. Diverticulectomy with or without resection of the part of the adjoining segment of intestine is required depending upon the intraoperative findings. In patients undergoing abdominal surgery with incidental finding of Meckel’s diverticulum, we suggest prophylactic resection of the diverticulum if it is long, has narrow base, adhered to the adjacent abdominal wall or mesentery. But the evidence for the prophylactic resection of Meckel’s diverticulum is lacking.

In conclusion, in patients with acute intestinal obstruction without previous abdominal surgery, Meckel’s diverticulum and its complications should be suspected. Abdominal CT scan is of great importance for the etiologic diagnosis and treatment of intestinal obstruction. Because Meckel’s diverticulum in low incidence, clinical preoperative diagnosis is very difficult. Once any complication occurs, it mostly results in surgical emergency.

## Data Availability

Not applicable.

## Abbreviations

BMI: Body mass index
CT: Computed tomography
